# Overcoming Barriers to Accessing Surgery and Rehabilitation in Low and Middle-Income Countries: An Innovative Model of Patient Navigation in Nepal

**DOI:** 10.1007/s00268-021-06035-1

**Published:** 2021-04-23

**Authors:** Jennifer L. Ibbotson, Bijata Luitel, Bikash Adhikari, Kathryn R. Jagt, Erik Bohler, Robert Riviello, Geoffrey C. Ibbotson

**Affiliations:** 1 Samaritan’s Purse Canada, 20 Hopewell Way NE, Calgary, AB Canada; 2 Sundar Dhoka Saathi Sewa, Bagdole – 4, PO Box 8975, EPC1961, Lalitpur, Nepal; 3 University of Oslo, Åsterudvn. 30, 1344 Haslum, Norway; 4 Department of Global Health and Social Medicine, Massachusetts Center for Surgery and Public Health, Brigham and Women’s Hospital, Harvard Medical School, Boston, MA USA; 5 United Nations Institute for Training and Research (UNITAR), Palais des Nations, 1211, Geneva 10, Switzerland

## Abstract

**Background:**

Injury and disability are prominent public health concerns, globally and in the country of Nepal. Lack of locally available medical infrastructure, socioeconomic barriers, social marginalization, poor health literacy, and cultural barriers prevent patients from accessing surgical and rehabilitative care. Overcoming these barriers is an insurmountable challenge for the most vulnerable and marginalized, resulting in absence of treatment or even death.

**Methods:**

Sundar Dhoka Saathi Sewa (SDSS), a non-government organization, provides a patient navigation service which facilitates referrals to tertiary centers from Nepal’s most remote areas. Specific criteria ensure that patient referrals are appropriate in regard to clinical and socioeconomic need, while comprehensive counselling helps guide the patient and family. The SDSS staff meet patients upon arrival in Kathmandu and facilitate admission to the appropriate tertiary hospital. They advocate for the patient, provide medicine, supply food and cover all treatment costs.

**Results:**

This project has enabled access to treatment for more than 1200 children for conditions leading to long-term disability and/or congenital heart disease. Interventions include a wide range of surgical and rehabilitative procedures such as complex orthopedics, cleft lip and palate, congenital talipes equinovarus, burn contractures, neurological cases, and cardiac surgery for valvular disease, septal defects and other congenital malformations.

**Discussion:**

The SDSS model of patient navigation is effective in overcoming the barriers to access surgical care and rehabilitation in Nepal. The success is owed to committed international donors, capacity building, effective counselling, advocacy, compassion, and community. We believe that this model could be replicated in other LMICs.

## Background

The World Health Organisation (WHO) recognizes that injury and disability are prominent global public health concerns. Traumatic injury alone causes more fatalities than malaria, TB and HIV combined [1, 2]. Of those who survive their injuries, many then suffer from significant disability as a direct result of delayed, non-existent or inadequate access to surgical management and rehabilitation care.

More than 1 billion people live with some form of disability [1, 3], with nearly 200 million experiencing considerable difficulties in functioning [1, 4]. The vast majority reside in low- and middle-income countries (LMIC), where disability is a key cause and consequence of poverty [3–5].

The United Nations Sustainable Development Goals (SDGs) promote health as a primary focus in Goal 3, “Ensure healthy lives and promote well-being for all at all ages” [6], which is closely linked to Goal 1: “End poverty in all its forms everywhere”. In order to achieve this, access to surgical care and rehabilitation must become a higher priority [7, 8]. The World Bank, the Lancet Commission on Global Surgery, and the United Nations have called upon all countries to ensure that no one is forced into poverty due to the costs of medical care [7–10].

Multiple barriers prevent patients in LMICs from accessing treatment: lack of locally available medical infrastructure; equipment; and clinical expertise; socioeconomic barriers; social marginalization; poor health literacy; and cultural barriers [3, 4, 11].

The country of Nepal faces significant challenges being ranked as one of the poorest countries in Asia (Human Development Index score 144/188). Its remote communities are considered some of the most vulnerable in the world [12–14]. With over 28 million people in an area covering 147,181km^2^, its population monograph recognizes 123 languages and more than 100 people groups [15], stratified into 125 identified castes [16].

Prior to 1950, Nepal was completely isolated from the outside world. The government has made great strides forward in improving healthcare since then. Today, they are working to deliver Basic Healthcare Services in an effort to reach Universal Health Coverage [17].

Presently, Nepal has eight central tertiary care hospitals, 12 regional level hospitals, 70 district level hospitals, nearly 13,000 primary healthcare outreach clinics, two rehabilitation centres, and one specialized Hospital and Rehabilitation Centre for Disabled Children (HRDC) [18, 19]. Although Nepal’s medical colleges graduate over 2000 physicians and 500 specialists annually, the majority remain in urban centres [20]. The availability of a surgeon at the district hospitals is variable with the remote facilities facing regular absenteeism [21].

In remote locations where advanced surgery or rehabilitation services are not available, the only option for receiving care is referral to Kathmandu [22–25].

Having worked for decades in isolated clinical settings, our team of clinicians and managers experienced repeated failure when referring patients because the most marginalized and vulnerable were unable to overcome the aforementioned barriers, resulting in the absence of treatment, sometimes even death.

## Method

In 2005, an indigenous Nepali church, whose many persons with disabilities knew first-hand the challenges of accessing healthcare in Kathmandu, initiated a comprehensive and integrated patient navigation service to overcome the barriers they had experienced. As a result of this work, a non-government organization called Sundar Dhoka Saathi Sewa (SDSS, Beautiful Gate Friend Service) was established in 2008.

SDSS identifies and provides solutions for accessing healthcare for vulnerable patients from Nepal’s most remote areas. For the purposes of this paper, we will specifically describe the Child Heart and Rehabilitation Care Project (CHRCP) from 2006 to 2018, hereafter called “the project”, one of several projects that SDSS manages.

The project’s initial goal was to enable remote hospitals and clinics to accept complex pediatric cases who require long-term rehabilitation, including surgical interventions, by giving them access to referral to a tertiary centre.

A year later, the criteria were expanded to include patients who required heart surgery for congenital or rheumatic heart disease. This part of the project integrates into the Government of Nepal’s free cardiac care program.

Additionally to navigating the patients through the referral process, the project provides funding for travel, food, and lodging for the patient and one caregiver. It offers financial coverage for rehabilitative surgery, orthotics, prosthetics, physical therapy, occupational therapy, speech and language pathology, casting, assistive devices, and pre-operative investigations. The financial support spans the entire referral process from the sending facility to the receiving facility and the child’s return home. Approximately 95% of the patients referred are considered extremely vulnerable and require full financial assistance. Funding from an international organisation has been constant since 2006, while always seeking to empower Nepali families to use their own finances whenever possible.

## Project criteria

Specific inclusion criteria ensure that patient referrals are appropriate with regard to clinical and socioeconomic need (Table 1). These criteria have played a critical role in the success of the project by defining the target patient groups and focussing the limited funding and manpower on specific clinical diagnoses with measurable outcomes.

**Table 1:** Project acceptance criteria

*Acceptance criteria*
1) Clear diagnosis of a physical disability or heart defect
2) Patient appropriate for extensive rehabilitation and/or surgical intervention
3) Patient under the age of 18
4) Completion of a standardized clinical assessment form by an approved rehabilitation therapist or physician confirming appropriateness of referral
5) Completion of a standardized socioeconomic assessment form by the sending hospital social services department, confirming socioeconomic need

The acceptance criteria are dependent on the patient’s condition with regard to diagnosis, appropriateness and age (1–3). They are dependent on the sending clinic with regard to assessment by a licensed rehabilitation therapist or physician, as well as assessment by social services (4–5)

To build sustainability, physicians, rehabilitation therapists and social workers at the sending facilities refer patients directly to the patient navigation centre at SDSS using a simple, step-by-step protocol (Table 2), which they have been trained to complete. Pediatricians skilled in cardiac ultrasound techniques hold the sole authority to complete the cardiac assessment.

**Table 2:** A simple, step-by-step protocol for the referral of rehabilitation patients

**Sending facility**
Rehabilitation therapists and/or physicians determine the need for long-term rehabilitation therapy - Complete clinical assessment form
Social services evaluate socioeconomic need - Complete social assessment - Contact the patient navigation centre by phone to initiate referral - Arrange transportation, food and lodging for one overnight in the bazaar (local town) if necessary - Communicate patient’s name, diagnosis and arrival time to the centre and to the rehabilitation coordinator via e-mail
**SDSS patient navigation**
Patient navigation (PN) staff meet patient and caregiver at the arranged arrival location (bus park, airport)
PN staff take patient and caregiver to the centre or directly to the hospital when urgent admission is needed: - Provide food and lodging
PN staff counsel the patient and family, requesting consent prior to admitting the patient into the project
PN staff accompany the patient to the hospital on the scheduled admission date: - Facilitate admission - Advocate for treatment - Assist in purchasing medicine and materials - Arrange inpatient food provision
PN staff visit regularly during admission
PN staff assist with discharge from the receiving facility
Healthcare coordinator evaluates patient’s mobility post-treatment, writes a discharge summary and sends it to the sending facility
PN staff arrange for follow-up treatment
PN staff arrange return travel for patient and caregiver and coordinate adaptations to home environment, when indicated

We chose sending facilities based on their remote location, a proven track record of providing high quality healthcare to the most marginalized people groups and their non-refusal policy.

## Referral process

Both the medical team and social services at the sending facility provide comprehensive counselling to the patient and family to explain the reason for the referral, including the estimated length of treatment and the expected outcomes. The social services staff contact the SDSS patient navigation coordinator in Kathmandu to authorize the referral.

**Fig. 1:**
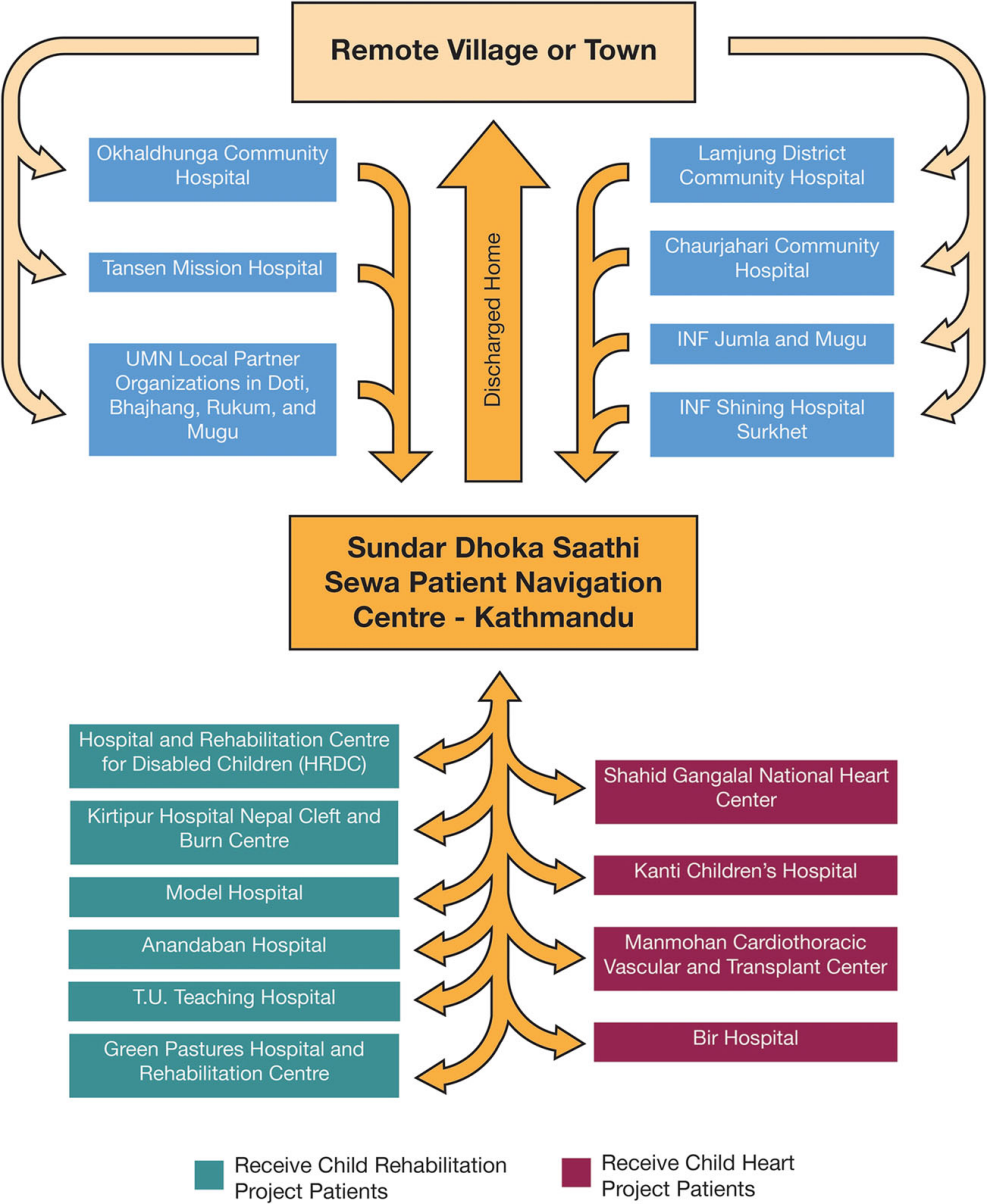
Patient flow chart

The patient navigation staff meet the patient and accompanying family member as soon as they arrive in Kathmandu and transport them either to the patient navigation centre or directly to the hospital if necessary (Fig. 1).

Patient navigators facilitate the process of hospital admission, advocate for appropriate attention from the medical staff, and counsel the patient and family. They assist the hospital staff with obtaining consent for treatment, purchase any medicines and materials required, supply nutritious food, and cover all costs associated to treatment. They regularly visit the patient throughout their admission and coordinate their discharge from the hospital. If the treatment includes outpatient care, they provide lodging, food and transport through the patient navigation centre. Finally, they organize the patient’s return home, educating the patient about follow-up visits and the care plan.

## Results

During the initial years, patient numbers started small, with only 17 patients referred in 2006–07. Referrals increased progressively during the following five years.

**Fig. 2:**
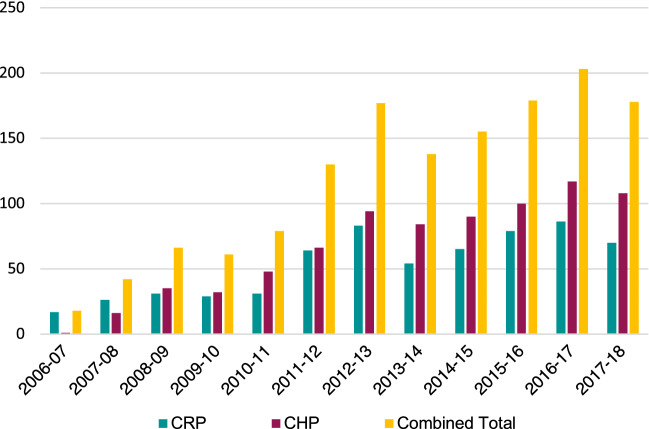
Annual number of CHRCP patients

The annual number of patients showed a sharp increase in early 2013 and again in early 2017 (Fig. 2). The first rise corresponded to the opening of the new patient navigation centre in Kathmandu, which increased the patient capacity of the project from 25 to 40 in-patients.

The second increase in 2016–17 corresponded to SDSS hosting a partner organization conference that brought together all the sending facilities and receiving tertiary centres. Clarifying criteria gave partners confidence to effectively focus their referrals.

Referrals increased when sending facilities developed their diagnostic abilities. Conversely, referrals dropped when sending facilities increased their capacity for treatment, for example, by hiring a physical therapist or an orthopedic surgeon, enabling patients to access treatment locally.

**Fig. 3:**
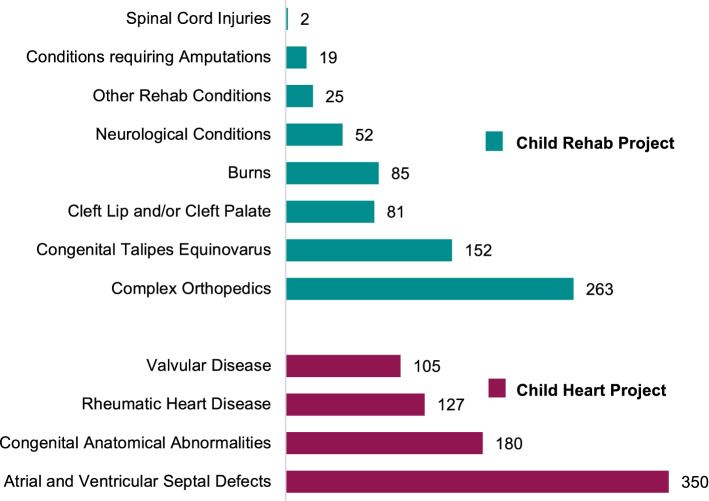
Patient load per diagnosis category 2006–2018

The rehabilitation component of the project received patients with burn contractures; cleft lip and/or palate; congenital talipes equinovarus (CTEV); complex orthopaedics; conditions requiring amputations; neurological disorders, and spinal cord injuries. Children with cardiac conditions presented with atrial and ventricular septal defects; valvular disease, complications of rheumatic heart disease; and congential anatomical abnormalities such as tetralogy of Fallot, Ebstein’s anomaly, patent ductus arteriosis, transposition of the great arteries, and truncus arteriosis (Fig. 3).

The rehabilitation surgical interventions included open reduction internal fixation (ORIF); external fixation; corrective osteotomy; amputation; stump revision; wound debridement; skin grafts; post-burn contracture release; heel cord release; tendo-Achilles lengthening; and spinal surgery including laminectomy and vertebral fusion. Ponsetti serial casting followed by splinting and orthotics was widely used for CTEV cases. All rehabilitation patients received comprehensive physical therapy, occupational therapy, and speech and language pathology.

The cardiac surgical interventions included septal defect closures, PTFE patch catheterization, intra-cardiac repair of tetralogy of Fallot, multiple techniques for valve repair and valve replacement.

These examples illustrate the complexity of surgical care accessed through the project, which would have otherwise been inaccessible (Table 3). Patients were often in critical condition due to extreme delays in diagnosis and treatment.

**Table 3:** Number of patients per treatment category

**CHP**	
Septal defect surgery	42
Surgery for other anatomical abnormalities	42
Valve surgery	30
Cardiac catheterization (diagnostic)	15
Medical treatment	298
**CRP orthopedic surgery**	
ORIF	57
Corrective osteotomy, bone graft, sequestrectomy	31
External fixator	22
Amputation, stump revision	21
Joint surgery (ankylosis release, arthrodesis, arthroplasty)	11
Alveolar bone graft	7
Skeletal traction	6
Implant surgery	5
Spinal surgery	2
**CRP soft tissue surgery**	
Heel cord release	63
Contracture release	42
Skin grafts (FTSG, STSG)	32
Surgical wound debridement, incision and drainage of abscess	29
Reconstructive surgery	25
Cleft lip repair	23
Palatoplasty	20
Tendo-Achilles lengthening	13
Neurological (craniectomy, myelomeningocele reduction)	5
**CRP conservative treatment and medical**	
Physical therapy	575
Occupational therapy	261
Prosthetics and orthotics	196
Casting (Ponsetti serial casting, single casting, hip spica, etc.)	145
Speech and language pathology	62
Assistive technology: walking devices, wheelchairs	8
Medical treatment	31

The numbers reflect single interventions, except for casting, occupational therapy, physical therapy, and speech and language pathology

The typical length of stay at SDSS in 2017–18 for patients coming for rehabilitation medicine was 40 days, compared to 13 days for heart patients.

The project began with three patient navigators. It has now grown to involve 32 staff, of whom 18 are directly engaged in patient navigation.

**Fig. 4:**
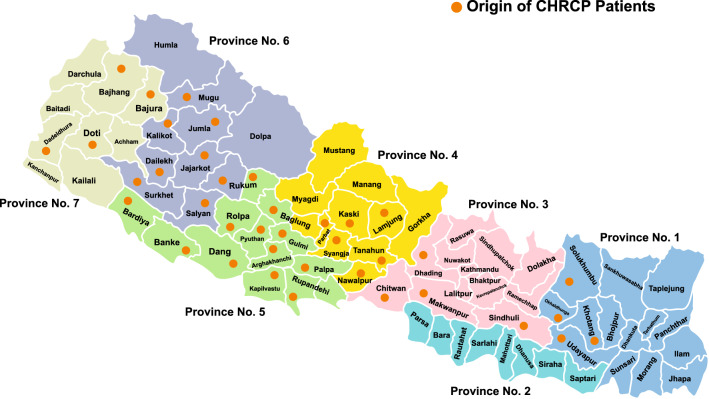
Origin of CHRCP patients

**Fig. 5:**
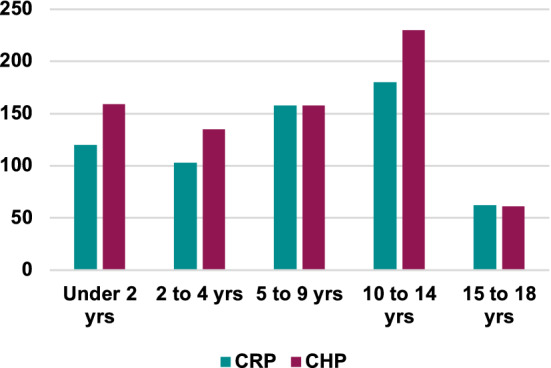
Age distribution of patients over the 12-year study period

Children from 38 of Nepal’s 77 districts came through the project (Fig. 4) with ages ranging from newborn to 18 years (Fig. 5).

## Discussion

We have found this model of patient navigation to be effective in overcoming the multiple barriers to access surgical care and rehabilitation in Nepal. As to be expected, this project faced a number of challenges (Table 4).

**Table 4:** Recurring challenges and solutions

Challenges	Solutions
Patient adherence	Patients show initiative by setting out to access the referring hospital for care If the patient arrives as an outpatient, the patient and caregiver are advised to immediately set off for Kathmandu the next day, avoiding a return home. If they return home to settle their affairs, they often do not return. The multidisciplinary team emphasizes the need to act quickly, avoiding any unnecessary returns to the village If the patient is coming from inpatient care, the referral is planned in advance and the family receives prior counselling
Loss of income	Flexibility is given as to who might be a caregiver: a grandparent, aunt/uncle, or sibling can accompany the patient to Kathmandu so that the parent can stay to provide income for the family or care for other children
Legal implications	Patient consent for navigation is obtained upon arrival at the PN centre
Mental health challenges related to disability	Integration of persons with disabilities (PWD) into key roles within the organisation Involvement in community life at SDSS gives birth to hope after disability
Blood shortage	Initiation of blood donor campaigns and establishment of a donor roster to assist the cardiac hospitals
Return to home village	Adaptation of home environment through local NGO

The following features contribute to reaching the goal of overcoming barriers:

## Committed partners

The greatest challenge is socioeconomic in nature. In addition to medical costs, patients face many hidden expenses related to transportation, lodging, food, and loss of income [4, 11]. Without financial assistance, many families will choose not to pursue treatment, thus prolonging the burden of disease and perpetuating the cycle of poverty.

Because the project focuses on vulnerable families, it is necessary for the cost of the program to be provided by an external charitable organization to guarantee sustainability. Despite having a sustained funding stream over the past 15 years, SDSS has diversified their income sources and projects beyond the CHRCP to strengthen their sustainability, including engagement with the local and national government services. Additionally, the SDSS staff have been in discussion with the Nepali Ministry of Health to explore ways of building the sustainability of the program by integrating patient navigation into the national health strategic plan.

## District level capacity-building

District hospitals serve a vital role in a country’s healthcare, despite this role having oftentimes been neglected. Empowering physicians improves interfacility referrals, a key factor in reducing delays in accessing care [26–28]. This project aims to encourage capacity building at the district level in rehabilitation medicine and pediatric cardiology through bridging the gap between remote facilities and tertiary care centres.

## Counselling

In remote areas, the absence of locally available medical infrastructure forces people to travel despite dangerous conditions or to forego the required treatment altogether [11]. Travel occurs only once all local resources have been exhausted, including visits to traditional healers and health posts, and after desperate agrarian families have sold land and livestock to cover previous treatment. Most families travel on foot to reach the sending facility, with their child suspended in a sling, or carried on a family member’s back [29]. They believe the remote clinic will be the last stop for definitive care. Hearing they are required to travel further to Kathmandu presents an insurmountable burden. Effective counselling at the sending facilities is essential to convince families to accept additional travel to receive care [28].

Patients from vulnerable people groups commonly have less access to education and therefore cultural beliefs, local traditions, and opposing advice from traditional healers impact their access to medical care [11]. Overcoming health illiteracy is complicated and multifactorial. It requires extensive individual counselling.

Counselling also plays an important role in accepting life-altering surgery. The patient navigators request a second opinion when medical recommendations do not match previous experience. This proactive advocacy has avoided several cases of unnecessary amputations.

Over time, the severity of patient conditions being admitted to the project increased. The patient navigators became adept at helping families understand when no further treatment was possible, assisting them through heart-breaking disappointment and encouraging them to accept a potential long-term disability. Occasionally, a patient in critical condition passed away. Patient navigators provided tremendous support to grieving families by walking through the tragedy alongside them, assisting with legal paperwork, initiating funeral procedures, and securing transportation home.

## Advocacy and expertise

Social marginalization is particularly evident when patients from remote villages or low social status must travel to a large metropolitan city, where illiteracy and language differences add further barriers to accessing care within the chaos of an overcrowded and sometimes dangerous environment. Patients and families from higher social classes tend to confidently demand and obtain services, whereas people from lower social classes and rural areas generally lack these skills [30, 31].

Interestingly, in situations when financial burden is not a problem, cultural barriers can still prevent access to care [11] and require advocacy on the part of the SDSS staff.

SDSS’ patient navigators have become experts with the hospital policies and procedures, granting them access to a variety of support programs offered by the government and other NGOs. They maintain current understanding of the services available at the multiple Kathmandu hospitals. Medical staff located at remote sending hospitals rely on the patient navigators’ knowledge, consulting them before making a formal referral.

## Compassion and community

SDSS has a no-refusal policy. This impacts the overall budget of the organization, while giving them a reputation for being compassionate. Indeed, the Kathmandu tertiary hospitals have grown to respect the SDSS services and often call upon them to assist with patients already admitted through other channels.

Nepal still grapples with significant stigma and prejudice surrounding disability. Many of the SDSS staff are persons living with disabilities, and therefore can demonstrate the potential for a positive future, offering hope to families who are struggling with a new diagnosis of permanent disability. Patients and their families develop supportive relationships within the SDSS community both during their stay and over scheduled follow-up visits.

The SDSS patient navigation model is unique in that it spans the country and functions as an interfacility system, linking remote sending facilities to a variety of specialized tertiary care facilities through a central patient navigation hub.

Present plans for expansion involve improving early intervention at the community level by accepting referrals from community-based rehabilitation facilities for select diagnoses.

## Conclusion

The comprehensive patient navigation model at SDSS is effective in overcoming many of the barriers faced by vulnerable patients in accessing appropriate tertiary facilities. It bridges the gaps in the existing healthcare system.

As humanity faces the new challenge of lockdowns to control COVID-19, the added barriers to access routine healthcare will inevitably lead to increases in morbidity and mortality [32, 33]. More than ever before, countries must maintain a high priority in assisting the most vulnerable as we strive to deliver Universal Health Coverage.

Patient navigation through an efficient interfacility referral system is an innovative strategy to reach some of the most remote and poverty-stricken places on earth. We believe that replicating this model would benefit patients in overcoming barriers to access surgical care and rehabilitation in other LMIC settings.

## Declaration

### Extra Web Material

For more information on the patient navigation program at Sundar Dhoka Saathi Sewa: https://vimeo.com/23621796
